# Domestic Remedy for Felons

**Published:** 1880-09-20

**Authors:** T. C. Brannon

**Affiliations:** Texas


### DOMESTIC REMEDY FOR FELONS.

BY T. C. BRANNON, M.D., TEXAS.

I have been afflicted with nearly forty felons during my life, and suffered very much with the first ones, being able to get no reliable treatment from medical authorities. In my desperation I resorted to the many treatments kindly suggested by old ladies, and finally succeeded in learning how to abort the inflammation.

Felons are generally, if not always, caused from bruises, and originate under the periosteum.

*How to diagnose a felon.*—When the palmar surface of the finger, thumb, or any part of the hand feels as if a fine, sharp, short thorn had entered the cuticle, and the outer end had become embedded beneath it, and when on "picking" for it it seems to be pressed into the periosteum, endways, and you fail to find the thorn, and know no other cause for redness, swelling and pain, you may rest assured that you have a felon coming. But if you wish to further satisfy yourself whether or not it is a felon, take your pocket knife and rub the edge over the small red spot, inclining the back of the blade forward. Notice if, when you are passing over the diseased spot, the red corpuscles are all caused to pass on through the blood vessels, leaving the inflamed part whitened for a short time after the knife passes over it. If not it is apt to prove to be a felon.

I give rules for diagnosing the disease in its forming stage, when it is easily aborted by my treatment; but if neglected longer, it only succeeds in part, according to the deposit under the periosteum.

*Treatment.*—I have used the following simple treatment for twenty-three years, since which I have always succeeded in aborting this painful disease, or modifying the great pain, and not unfrequently preventing the loss of one joint of the finger:

Take of soft lye soap and flaxseed meal a sufficient quantity, stirring the meal in slowly with spatula, or case knife, manipulating thoroughly, so as to form a salve or poultice. Cornmeal is a good substitute for the flaxseed. Envelop the finger in this, applying snuggly, and occasionally pressing it to bring it more completely in apposition. Renew the poultice every twelve to twenty-four hours.

Don't try every prescription you may hear of. Depend on this, and this alone. It will, if applied in time, abort the disease; if adopted later, it will bring it to a small "head" (if too far advanced to be "scattered") when it may be picked almost painlessly.

The escharotic properties of the soap soon destroy the thick skin over the region of the disease, which accounts partly for the quick relief from pain. Besides I think it is partially absorbed, and thus affects, more or less, the diseased process.—*Ther. Gazette.*

